# Assessment of Effects of Rosemary Essential Oil on the Kidney Pathology of Diabetic Adult Male Albino Rats

**DOI:** 10.7759/cureus.35736

**Published:** 2023-03-03

**Authors:** Shimaa A Fareed, Einas M Yousef, Samar M Abd El-Moneam

**Affiliations:** 1 Human Anatomy and Embryology, Faculty of Medicine, Suez Canal University, Ismailia, EGY; 2 Histology and Cell Biology, Faculty of Medicine, Menoufia University, Shibin El Kom, EGY

**Keywords:** pcna, bax, streptozotocin, diabetic nephropathy, antioxidants, rosemary essential oil

## Abstract

Background

Diabetic nephropathy is a severe condition that causes persistent kidney problems and chronic renal failure. Rosemary (Rosmarinus officinalis L) is widely recognized for its antioxidant, antidiabetic, anti-inflammatory, antithrombotic, hepatoprotective, and anticancer activities. The current study evaluated rosemary essential oil (REO) effects on biochemical, histological, and immunohistochemical kidney alterations in streptozotocin (STZ)-induced diabetic rats and compared these effects with those of insulin and both combined.

Methods

We randomly distributed 36 adult albino rats into 6 groups: normal control (non-diabetic), diabetic (streptozotocin, 55 mg/kg, intraperitoneal), diabetic insulin-treated (Lantus insulin 2 units/day, SC), diabetic REO-treated (REO, 10 ml, nasogastric gavage), and diabetic insulin & REO-treated groups. Biochemical, histological, and immunohistochemical analyses were conducted.

Results

The diabetic group revealed a substantial increase in blood glucose, urea, creatinine, and uric acid, as well as malondialdehyde (MDA) and catalase (CAT) concentrations in kidney homogenates, high score of tubular injury, and increased glomerulosclerosis, along with marked reduction of total glutathione (GSH) and superoxide dismutase (SOD) when compared to control. Evident improvement was detected in rats treated with REO as it demonstrated antioxidant, anti-inflammatory, anti-apoptotic, pro-proliferative, and mild anti-hyperglycemic effects on diabetic rats, reducing the kidney damage caused by diabetes. Combined insulin and REO restored normal blood glucose, renal excretory function tests, antioxidant markers, and renal cortex histology.

Conclusion

The data presented in the current study's in vivo animal model suggests that REO supplementation has beneficial nephroprotective effects on the structural and, to a lesser extent, functional levels of diabetic rats. Furthermore, the detected nephroprotective effects of insulin and REO combined are superior to those of either administered alone. However, further studies are needed to evaluate these conclusions in humans further.

## Introduction

Diabetes mellitus (DM) is a chronic metabolic disorder characterized by diminished insulin secretion or action resulting in hyperglycemia with subsequent complications in many organs such as the heart, retina, nervous tissue, and kidney. According to the International Diabetes Federation, over 80% of end-stage renal disease (ESRD) worldwide is caused by diabetes, high blood pressure, or a combination [1]. Diabetic nephropathy (DN) is a serious consequence of DM that causes persistent kidney problems and ESRD [2]. DN is characterized by kidney structural abnormalities, including glomerulosclerosis, renal fibrosis, and thickening of the basement membranes of various renal compartments [3]. Furthermore, it is presented clinically by persistent proteinuria and progressive decline of glomerular filtration rate with subsequent progressive renal failure in diabetic patients [4].

The pathogenesis of DN is multifactorial that involves many pathophysiological processes and molecular pathways. Persistent hyperglycemia is one of the primary factors of kidney injury and subsequent oxidative stress in diabetic patients [5]. A high concentration of reactive oxygen species (ROS) activates several molecular pathways that impair the antioxidant defense system, resulting in DN [6]. There is growing evidence suggesting that inflammation is crucial in the pathogenesis of DN. Numerous pro-inflammatory molecules affect glomerular functions by altering vascular permeability, inducing the proliferation of various glomerular components, and disturbing the extracellular matrix dynamics [7]. Another crucial factor in the pathogenesis of DN is glomerular hyperfiltration which is believed to be a sign of elevated intraglomerular capillary pressure [8]. Different forms of programmed cell death, such as apoptosis and autophagy, are recently identified to be implicated in the development of DN [7]. Current DN therapeutic regimens aim to delay disease progression; however, many controversies exist about their clinical efficacy and adverse reactions. Hence, novel strategies to treat DN or to supplement current therapeutic regimens are required.

The ubiquitous household herb known as rosemary, Rosmarinus officinalis L (Family Lamiaceae), is widely grown worldwide. Currently, rosemary oil extract is approved as a food additive in various food categories [9]. Recent research suggests that R. Officinalis plays a fundamental role in many organs' protection by exhibiting antioxidant, antidiabetic, anti-inflammatory, antithrombotic, hepatoprotective, and anticancer activities [10]. Rosemary has been used in traditional medicine to effectively ameliorate illnesses such as headaches, inflammatory disorders, stomach pain, and depression [11,12]. The European Medicines Agency recently suggested using Rosemary oil extract to treat dyspepsia, mild gastrointestinal spasms, minor muscle aches, and peripheral circulation disorders [13]. Few recent studies demonstrated the potential nephroprotective effects of Rosemary Essential Oil (REO) [10-12]; nevertheless, this is the first study to evaluate its influence on the histological alterations of streptozotocin (STZ)-induced diabetic nephropathy. Therefore, this study aimed to evaluate the effects of REO, insulin, and both combined on the kidney's biochemical, histological, and immunohistochemical alterations in STZ-induced diabetic rats.

## Materials and methods

Chemicals and reagents

Rosemary Essential Oil (REO), 25 ml, was purchased from Nefertari Body Care Products Manufactory. The following items were purchased from Sigma Chemical Co Analytical: Streptozotocin (STZ), Lantus insulin (insulin glargine injection), and 1- Diphenyl-1-picrylhydrazyl (DPPH) dark-colored crystalline powder (25gm). 

1,1-diphenyl-1-picrylhydrazyl (DPPH) free radical scavenging test for the evaluation of in vitro antioxidant activity

Using Rašković et al. protocol, a new DPPH solution was prepared by mixing 2mg of DPPH with 100 ml of ethanol in test tubes, and serial dilutions of REO were added to each test tube [14]. The reaction mixture was vortexed vigorously and allowed to rest for 30 minutes at room temperature in the dark. A UV spectrophotometer was used to measure the absorbance at 517 nm. Ascorbic acid was employed as a positive control and was dissolved in distilled water to achieve the same concentration as REO in the stock solution. The IC50 value was determined and represented in μl of essential oil per ml, which indicates the sample concentrations necessary to scavenge 50% of the DPPH free radicals. All tests were performed in triplicate, and the average values were used. 

Animals 

During this study, thirty-six healthy adult male Sprague-Dawley albino rats, aged 2 months, ranged in weight from 140 to 160 grams. Animals were obtained from the Suez Canal University, Faculty of Veterinary Medicine's animal house. The animals had access to regular rat chow and water and were kept in a typical light-dark cycle at room temperature. They were kept for ten days before being subjected to any experimental procedures. This study was carried out following the guidelines of the Research Ethics Committee of the Faculty of Medicine, Suez Canal University (Egypt) (Research Number: 4858#) and was approved on May 15, 2022.

Experimental design

After a pilot study, a total of 36 animals were distributed into the following groups (No. = 6 per group or subgroup): group I (control group): non-diabetic rats which were divided into group IA (negative control group): rats received no medication and group IB (REO positive control group): over 8 weeks, rats received 10 mg/kg REO after being suspended in saline by nasogastric gavage daily [14]. A mixture of 1ml REO:99ml saline was used to ensure a constant dose of 10mg/kg, and then the mixture was administered to rats based on their weights. Group II (diabetic group): one intraperitoneal injection of STZ (55 mg/kg body weight) dissolved in citrate buffer (pH 4.7, 4.5 M) was administered to rats [15]. Diabetes was verified 72 hours later by testing fasting blood glucose levels using glucose-oxidase reagent strips (ONE TOUCH Ultra Easy). When blood glucose levels exceeded 200 mg/dL, rats were considered diabetic [16]. Group III (diabetic insulin-treated group): diabetic rats received 2 units of Lantus insulin daily (immediately after accurate estimation of hyperglycemia) through subcutaneous (SC) injection for 8 weeks [17]. Group IV (diabetic REO-treated group): over 8 weeks, diabetic rats received 10 ml of REO after being suspended in saline by nasogastric gavage daily. Group V (diabetic insulin & REO-treated group): both Lantus insulin (2 units/day, SC injection) and REO (10 ml/day, nasogastric gavage) was administered daily to diabetic rats.

Collection of blood and tissue samples 

Following 8 weeks of the experiment, the rats fasted overnight, and the blood samples (4 ml/rat) were collected from the retro-orbital plexus under light anesthesia. Blood samples were left to coagulate for 20 minutes, then centrifuged at 5000 rpm for 10 minutes, and the separated sera were stored at -20 °C for further biochemical tests. After collecting blood samples, the rats were euthanized by beheading under anesthesia using an intraperitoneal injection of thiopental (50mg/kg) [18]. The collected kidneys were split into two halves. One half was used to prepare the kidney homogenates, which were prepared by homogenizing 1 g of frozen kidney tissues with a Tris-HCl sucrose buffered solution in a 1:3 (w/v) ratio at 4 °C. The other half was immersed in neutral buffered formalin (10%) and routinely processed for further histological analyses. 

Serum biochemical parameters

Precision Xtra Plus test strips assessed random blood glucose levels in separated serum. Moreover, serum levels of urea, creatinine, and uric acid (mg/dl) were assessed using commercially available kits using the recommended spectrophotometric methods. All samples were analyzed in triplicate.

Oxidative stress markers

The levels of Malondialdehyde (MDA) and total glutathione (GSH) were assessed in the prepared kidney homogenates using the appropriate spectrophotometric techniques [19,20]. Superoxide dismutase (SOD) [21] and catalase (CAT) assays [22] were also performed to assess their activity. These tests detect the reduction in absorbance produced by NADPH oxidation at 340 nm (UV assay). Each sample was analyzed in duplicate, and the average readings of 6 samples were calculated for each group.

Histological studies

Fixation and staining were carried out following Suvarna et al. [23]. Sections of 5μm thickness were prepared using Leica RM2025 microtome and stained with Hematoxylin and Eosin (H&E). On H&E-stained sections, the percentages of renal tubules with dilatation (the number of the affected tubules/ total number of the counted tubules), atrophy, cellular vacuolations, cellular necrosis, and cast formation were determined to score the renal tubular damage histologically. On a scale from 0 to 5, the scoring technique was as follows: 0 = normal; 1 = 10%; 2 = 10-25%; 3 = 26-50%; 4 = 51-75%; and 5 = >75% [24]. Tubular injury scoring was evaluated in 10 randomly selected fields at a magnification of x400 using an Olympus BX-46 microscope equipped with an Olympus SC30 digital camera. 

Staining with Periodic Acid Schiff (PAS) was utilized to evaluate the mesangium, glomerular and tubular basement membranes, and brush borders of renal tubules. Furthermore, Toluidine blue staining was utilized to assess the nuclei of distinct components of the renal tissues, as it enhances the clarity of the nuclear features.

Immunohistochemical (IHC) study

Immunohistochemical analysis of the proliferating cell nuclear antigen (PCNA) and Bcl-2 Associated X-protein (BAX) was conducted on paraffin sections of kidneys from different study groups. PCNA (anti-PCNA, monoclonal mouse, dilution 1:100; DAKO, Glostrup, Denmark) and the anti-BAX (polyclonal rabbit, dilution 1:40; DAKO Corporation, Carpinteria, CA, USA) primary antibodies were used in this analysis. The immunohistochemical staining for BAX and PCNA was evaluated by two independent observers on a random five fields/slide by power field (x400) using a semiquantitative scoring method that includes two parameters as previously described by O'Hara et al. [24]. The first parameter is the percentages of immunoreactive cells (0 to 5), and the second is the intensity score (0 to 4). The sum of the two scores (ranges from 0 to 9) was considered the total score of the antibody expression.

Morphometric measurements

Image J software (version 1.53K) was used for quantitative morphometric assessment of kidney sections of different experimental groups (National Institutes of Health, USA) [25]. For each parameter, ten non-overlapping fields per section were randomly examined in each group (five animals/group). The morphometric assessment included the following: A) the diameter of the renal corpuscle was measured in H&E-stained sections (x400). B) The index of mesangial expansion was assessed in the PAS-stained sections (x400) using the following formula: (PAS positive area x 100/area of the corresponding glomeruli) [26].

Statistical analysis

Data were presented as mean ± standard deviation (SD). The intergroup variation was evaluated using a one-way variance analysis (ANOVA) and the Bonferroni post hoc test. When p < 0.05, the results were considered statistically significant. Data were analyzed using SPSS software, version 19 (IBM SPSS, Chicago, IL, USA).

## Results

In vitro antioxidant activity of REO 

REO's scavenging activity was assessed using the DPPH free radical scavenging assay compared to ascorbic acid as a positive control. The IC50 value of REO was 78.4 μl/ml, indicating its intense radical scavenging activity.

Random blood glucose level

**Figure 1:**
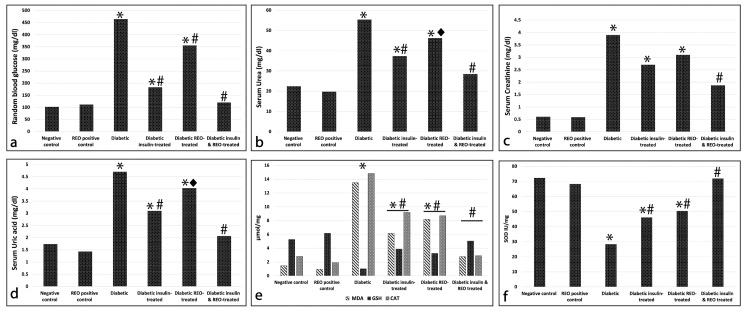
Effect of REO on (a) Random blood glucose (mg/dl), (b) serum urea (mg/dl), (c) serum creatinine (mg/dl), (d) serum uric acid (mg/dl), (e) MDA, GSH, and CAT oxidative stress markers (μmol/mg), (f) SOD oxidative stress marker (IU/mg). (*) significantly different from control groups; (#) significantly different from the diabetic untreated group, p < 0.01. (Diamond) significantly different from the diabetic untreated group, p < 0.05.

Our results revealed that diabetic rats showed significantly higher levels of random blood glucose when compared to control groups (control: 102.3 ± 6.59 mg/dl, diabetic: 464.3 ± 64.72 mg/dl, p < 0.001). Blood glucose levels were significantly decreased (p < 0.001) in diabetic rats treated with insulin (183.22 ± 20.31 mg/dl), REO (355.8 ± 56.85 mg/dl), or a combination of the two (120.3 ± 6.71 mg/dl) compared to the diabetic group. However, the administration of REO in diabetic rats was not able to restore normal blood glucose levels (Figure 1a). These findings indicate that the effect of insulin administration outperforms REO alone in lowering the blood glucose levels of diabetic rats. Moreover, combined insulin and REO were more effective in regulating blood glucose than administering either REO or insulin alone.

Biochemical parameters of the renal excretory function

Our findings demonstrated that the diabetic group showed significantly higher levels of serum urea (55.34± 12.02 mg/dl), creatinine (3.9± 1.32 mg/dl), and uric acid (4.7± 1.03 mg/dl) when compared with the control groups (p < 0.001). The diabetic groups treated either with insulin or REO showed a highly significant decrease in the level of serum urea (insulin: 37.32± 10.05 mg/dl, REO: 3.1± 1.03 mg/dl, p < 0.001) and a significant decrease in the level of uric acid (insulin: 3.1± 1.23 mg/dl, REO: 4.03± 1.76 mg/dl, p < 0.05) when compared with the diabetic untreated group. However, a highly significant (p < 0.001) increase in serum urea, creatinine, and uric acid levels was still detected in these two groups compared to the control. Simultaneous administration of insulin and REO induced a highly significant decrease in the level of the three assessed biochemical parameters (urea: 28.46 ± 3.17 mg/dl, creatinine: 1.87 ± 0.7 mg/dl, uric acid: 2.07± 0.39 mg/dl, p < 0.001) when compared with the diabetic group. Notably, the levels of serum urea and uric acid could not be normalized by the simultaneous administration of insulin and REO since a significantly high level (p < 0.05) was still observed in comparison to the REO control (Table 1) (Figure 1b-1d).

**Table 1:** The mean (± SD) level of random blood glucose and renal biochemical parameters in different experimental groups REO: Rosemary Essential Oil, Compared to ^a ^control, ^b ^REO control, ^c ^diabetic, ^d ^diabetic insulin-treated, ^e ^diabetic REO-treated, ^f ^diabetic insulin & REO-treated, *p < 0.05, **p < 0.01

Groups	Random blood glucose (mg/dl)	Urea (mg/dl)	Creatinine (mg/dl)	Uric acid (mg/dl)
Negative control	102.3 ± 6.59	22.44 ± 2.12	0.61 ± 0.22	1.74± 0.42
REO positive control	111.6 ± 3.15	19.74 ± 1.8	0.59 ± 0.42	1.43± 0.04
Diabetic	464.3 ± 64.72 ^**(a,b,d,e,f)^	55.34± 12.02 ^**(a,b,d,f), *(e)^	3.9± 1.32 ^**(a,b, f) ^	4.7± 1.03 ^**(a,b, d,f), *(e)^
Diabetic insulin-treated	183.22 ± 20.31 ^**(a,b,c,e,f)^	37.32± 10.05 ^**(a,b,c),*(e,f)^	2.7± 1.32 ^**(a,b)^	3.1± 1.23 ^**(a,b,c,e,f)^
Diabetic REO-treated	355.8 ± 56.85 ^**(a,b,c,d,f)^	46.22± 8.55 ^**(a,b,f,) , *(c,d)^	3.1± 1.03 ^**(a,b)^	4.03± 1.76 ^**(a,b,e ,f), *(c)^
Diabetic insulin & REO-treated	120.3 ± 6.71 ^**(c,d,e)^	28.46 ± 3.17 ^**(c,e) , *(b,d)^	1.87 ± 0.7 ^**(c)^	2.07± 0.39 ^**( c ,d,e) , * (b)^

Oxidative stress markers 

Our results revealed significantly higher levels of MDA (13.5 ± 4.03 μmol/mg, p < 0.001) and significantly lower levels of GSH (1.02± 0.02 μmol/mg, p < 0.001) in kidney tissues of diabetic rats than in the control groups. Insulin or REO administration significantly reduced the level of MDA in kidney tissues when compared with the diabetic untreated rats (insulin: 6.16 ± 1.05 μmol/mg, REO: 8.18 ± 2.22 μmol/mg, p < 0.001). Nevertheless, a significantly elevated (p < 0.001) level of MDA was identified in diabetic rats treated with insulin or REO when compared with the control groups. Furthermore, when insulin and REO were administered together, the level of MDA substantially reduced (2.8 ± 0.75 μmol/mg, p < 0.001) compared to the diabetic group and the other two treated groups. Notably, there was no considerable variance between the combined treated and the control groups. Alternatively, diabetic rats treated with insulin or REO demonstrated a significantly lower level of GSH when compared with the normal control (insulin: 3.88 ± 0.66 μmol/mg, REO: 3.26 ± 0.77 μmol/mg, p < 0.05) and significantly higher levels (p < 0.001) were detected when compared with the untreated diabetic rats. When treating the diabetic rats with both insulin and REO combined, the level of GSH significantly raised (5.04 ± 0.9 μmol/mg, p < 0.001) compared to the diabetic untreated group, approaching normal levels (Figure 1e). 

When assessing the activity of SOD and CAT, our findings revealed a highly significant decline in SOD (28.4 ±2.05IU/mg, p < 0.001) and a highly significant rise in CAT (14.83 ± 3.23 μmol/mg, p < 0.001) activity in a diabetic group when compared with the control groups. When compared to the diabetic group, diabetic rats treated with insulin or REO displayed a considerable increase (insulin: 46.11 ± 3.07 IU/mg, REO: I50.4 ± 5.02 U/mg, p < 0.001) in SOD activity and a significant decline (insulin: 9.21 ± 1.08 μmol/mg, REO: 8.71 ± 2.01 μmol/mg p < 0.001) in CAT activity. However, the activity of both enzymes remained significantly different (p < 0.05) from that of the control group. Combined insulin and REO administration markedly elevated the activity of SOD (71.9 ± 5.12 IU/mg). It decreased the activity of CAT (2.91 ± 0.98 μmol/mg), which showed near normal value (p > 0.05) (Table 2) (Figure 1e, 1f).

**Table 2:** The mean (±SD) levels of oxidative stress markers in kidney tissue of different experimental groups REO: Rosemary Essential Oil, MDA: Malondialdehyde, GSH: Total glutathione, SOD: Superoxide dismutase, CAT: Catalase. Compared to ^a ^control, ^b^REO control, ^c ^diabetic, ^d ^diabetic insulin-treated, ^e ^diabetic REO-treated, ^f ^diabetic insulin & REO-treated, *p < 0.05, **p < 0.01

Animal groups	MDA	GSH	SOD	CAT
	μmol/mg	μmol/mg	IU/mg	μmol/mg
Negative control	1.46 ± 0.31	5.28 ± 0.16	72.31 ± 3.42	2.81 ± 0.18
REO positive control	0.96 ± 0.21	6.18 ± 0.47	68.31 ± 4.02	1.92 ± 0.27
Diabetic	13.5 ± 4.03^**(a,b,d,f,e)^	1.02± 0.02^**(a,b,d,e,f)^	28.4 ±2.05^**(a,b,d,e,f)^	14.83 ± 3.23^**(a,b,d,e,f)^
Diabetic insulin-treated	6.16 ± 1.05 ^**(a,b,c,f), *(e)^	3.88 ± 0.66 ^**(b,c,f) *(a)^	46.11 ± 3.07 ^**(a,b,c,f)^	9.21 ± 1.08 ^**( a,b,c,f)^
Diabetic REO-treated	8.18 ± 2.22^**(a,b,c,f), *(d)^	3.26 ± 0.77 ^**(a,b,c,f)^	50.4 ± 5.02 ^**(a,b,c,f)^	8.71 ± 2.01^**(a,b,c,f)^
Diabetic insulin & REO treated	2.8 ± 0.75 ^**(c,d,e)^	5.04 ± 0.9^**^^(^^ c,e)^	71.9 ± 5.12 ^**^^(^^c,d,e)^	2.91 ± 0.98 ^**^^(^^c,d,e)^

Histological findings

H&E Staining

**Figure 2:**
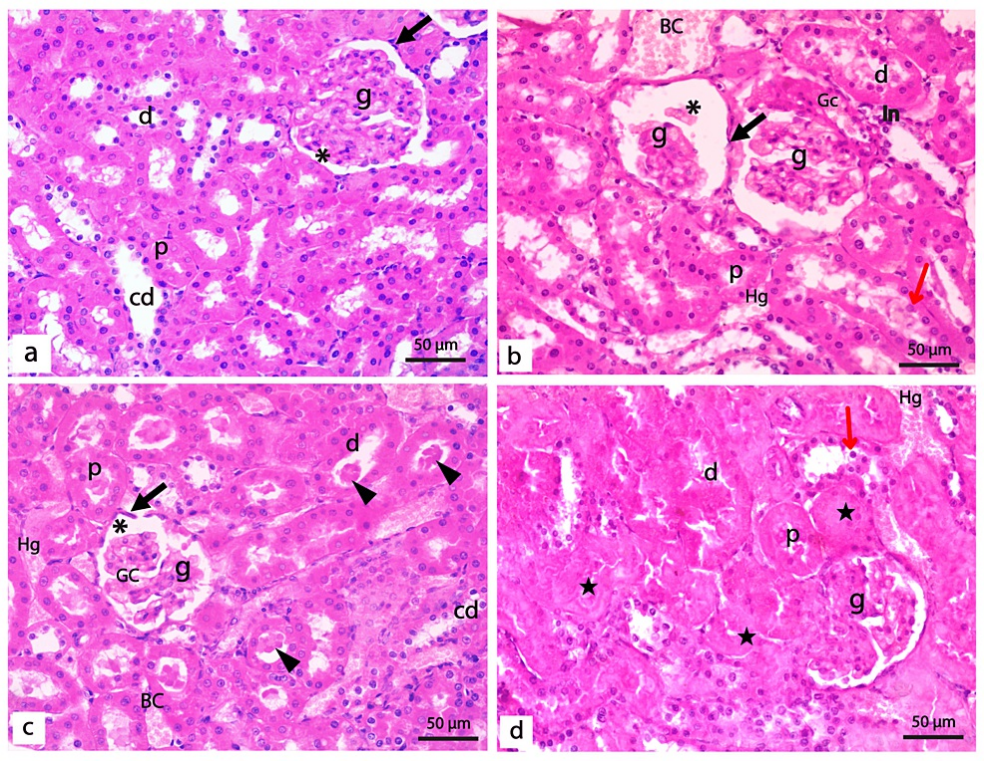
Photomicrographs of renal cortex of the control and diabetic untreated groups. (a) Normal control group showing normal renal corpuscles formed of a tuft of capillaries of the glomerulus (g) surrounded by Bowman′s capsule enclosing renal space (*). Bowman's capsule's parietal layer (black arrow) is lined by flattened epithelial cells. Proximal tubules (p) have a narrow lumen lined with pyramidal cells with acidophilic cytoplasm, basal rounded vesicular nuclei, and a well-developed apical brush border. The lining of the distal tubules (d) is cubical cells with light acidophilic cytoplasm and central rounded vesicular nuclei with a less developed brush border. In addition, collecting ducts (cd) are lined by simple cubical epithelium with central rounded vesicular nuclei. (b-d): sections of the diabetic group showing shrunken and distorted glomeruli (g) with congested glomerular capillaries (Gc). The glomeruli are surrounded by unpreserved Bowman's capsule (black arrow) lined by cells flattened nuclei with wide Bowman's space (*). Varying degrees of hydropic degeneration and coagulative necrosis (star) of cells lining the tubules where both proximal (p) and distal (d) tubules showed ill-defined lumens with deposition of the hyaline cast (arrowheads). Cells lining renal tubules showed loss of linear nuclear arrangement with shrunken, condensed, and exfoliated nuclei (red arrows) and loss of brush borders. Notice the area of periglomerular inflammatory infiltration (In), area of hemorrhage (Hg), and congested blood capillaries (BC) between the renal tubules. (H&E x400, bar 50μm).

Histologically, the negative and REO positive control groups showed no detectable differences. The renal cortex of the control rats demonstrated normal renal corpuscles constituted of glomeruli surrounded by the Bowman′s capsules and normal proximal and distal tubules (Figure 2a). In diabetic rats, shrunken and disrupted glomeruli enclosing wide and congested glomerular capillaries were detected. The glomeruli were surrounded by unpreserved Bowman's capsules and had wide Bowman's space. Both proximal and distal tubules of diabetic rats showed ill-defined lumens with deposition of the hyaline cast and many exfoliated nuclei. The cells lining the renal tubules showed varying degrees of hydropic degeneration, coagulative necrosis, and loss of brush borders. Areas of inflammatory infiltration, together with congested blood capillaries, were also detected between the renal tubules (Figure 2b-2d).

**Figure 3:**
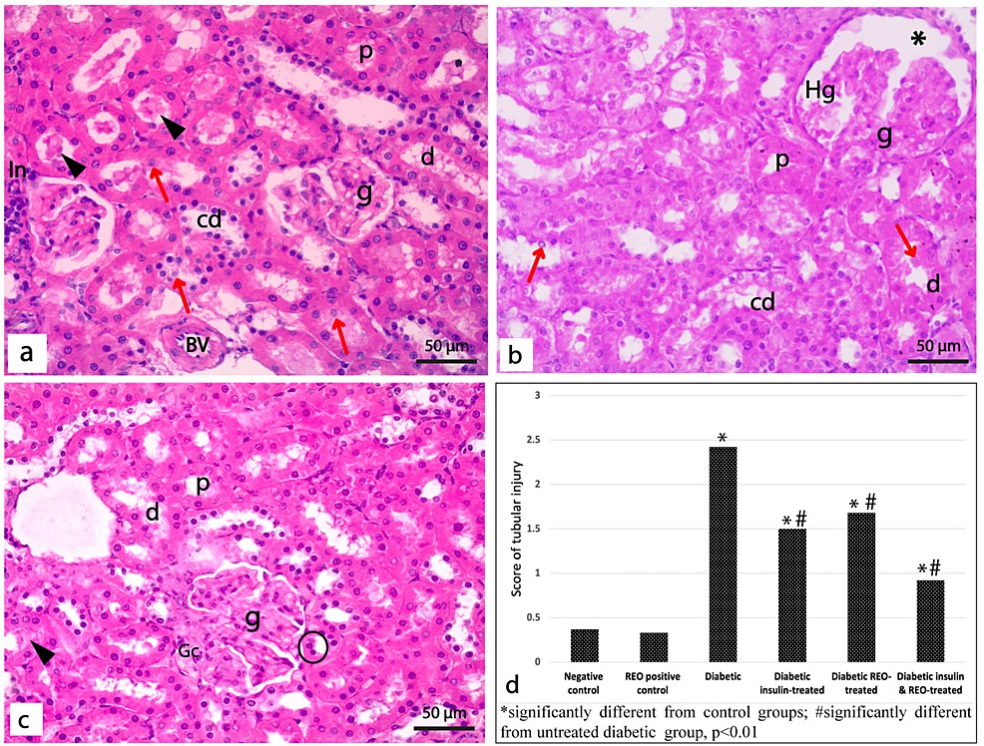
Photomicrographs of the renal cortex of treated groups. (a) Diabetic rats treated with insulin showed improved renal architecture; however, some distorted glomeruli (g), hyaline cast (arrowheads) in some proximal (p), and distal (d) tubules with desquamated nuclei (red arrows), and periglomerular inflammatory cells (In) are detected. (b) Diabetic rats treated with REO showed partial restoration of normal renal architecture. However, some renal corpuscles show disturbed glomerulus (g), wide renal space (*), and hemorrhage (hg). Most of the proximal (p) and distal (d) tubules are showing normal lining epithelium with the restoration of the brush border; however, few desquamated cells can also be detected (red arrows) (c) Diabetic rats treated with both insulin and REO showing a normal architecture of the kidney with normal glomeruli (g), however, some congested glomerular capillaries (Gc) can be detected. Some cells lining the proximal and distal tubules are binucleated (black circle), and a scanty hyaline cast (arrowhead) is detected in the lumen of a few tubules. (a-c: H&E x400, bar 50μm). (d) Tubular injury score in different experimental groups; (*) significantly different from control groups; (#) significantly different from the diabetic untreated group, p < 0.01.

Administration of REO or insulin was found to correct the histological aberrations detected in diabetic rats partially. In both groups, a larger proportion of glomeruli displayed normal structure; nevertheless, congested glomerular capillaries were still detected. A marked decrease in tubular necrosis, along with less hyaline cast deposition, was detected in both groups. However, the linear nuclear arrangement of some proximal and distal tubules was disrupted in both groups, with the sloughing of some nuclei inside the tubular lumen (Figure 3a, 3b). Interestingly, examining kidney sections from diabetic rats treated with REO and insulin demonstrated that the renal architecture was almost the same as in control. The glomeruli and renal tubules appeared almost normal, except for some congested glomerular capillaries and scanty hyaline cast (Figure 3c).

To further investigate the impact of REO on the kidney's histological architecture in STZ-induced diabetic rats, measurement of glomerular diameter and assessing the tubular necrosis in the H&E-stained sections were conducted. For glomerular diameter, a highly significant (p < 0.001) decrease was detected in the diabetic group when compared with the control groups. Although the administration of insulin, REO, and both combined increased the diameter of renal glomeruli, a statistically significant increase (p < 0.001) was only detected in the diabetic group treated with both insulin and REO when compared with the untreated diabetic group (Table 3). On the other hand, when assessing the tubular injury scores, a highly significant (p < 0.001) increase was detected in the diabetic group compared to the control groups. The administration of insulin, REO, and both combined induced a statistically significant decrease in tubular injury scores (p < 0.001) compared to the diabetic untreated group (Figure 3d) (Table 3).

**Table 3:** Mean ± SD of the assessed histological parameters in the glomeruli and renal tubules of different experimental groups REO: Rosemary Essential Oil, Compared to ^a ^control, ^b ^REO control, ^c ^diabetic, ^d ^diabetic insulin-treated, ^e ^diabetic REO-treated, ^f ^diabetic insulin & REO-treated, *p < 0.05, **p < 0.01

Animal groups	Glomerular diameter (μm)	Score of tubular injury	Index of glomerulosclerosis
Negative control	518.59 ± 92.54	0.37± 0.05	8.03 ± 1.25
REO positive control	515.58 ± 64.73	0.33± 0.08	8.00 ± 0.96
Diabetic	379.05 ± 106.63 ^**(a,b), *( f)^	2.42±0.34^**^^(a,b,d,ef)^	30.27 ± 9.35 ^**(a,b,d,e,f)^
Diabetic insulin-treated	452.52 ± 120.6	1.5±0.12^**(a,b,c, f)^	16.60 ± 4.43 ^**^^(a,b,c), *(f)^
				Diabetic REO-treated	494. 2 ± 57.49	1.68±0.09^**(a,b,c, f)^	13.42 ± 3.46 ^**( c)^
Diabetic insulin & REO-treated	500 ± 69 ^*(C)^	0.92±0.12^**^^(a,b,c,d,e)^	8.29 ± 2.04 ^**(c), *(d)^

Toluidine Blue Staining

**Figure 4:**
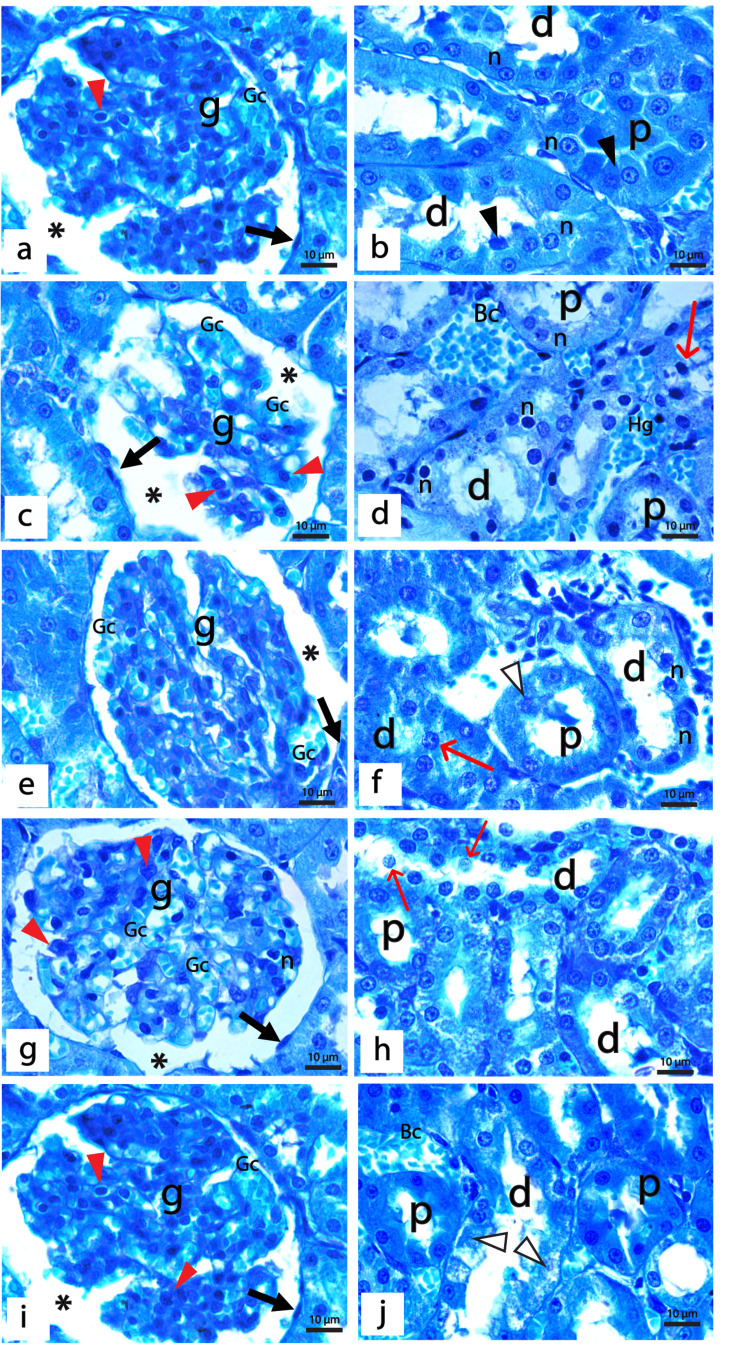
Photomicrographs of semithin sections of the renal cortex of different experimental groups. (a-b) Control groups showing (a) Glomerulus (g) lined by flattened cells (black arrow) enclosing glomerular capillaries (Gc) and mesangial cells (red arrowhead), surrounded by Bowman's space (*). (b) Proximal tubule (p) lined by pyramidal cells with rounded basal nuclei (n) and distinct brush border (arrowhead). Distal tubules (d) are lined by cubical cells with vesicular nuclei (n) and a less developed brush border (arrowhead). (c-d) Diabetic group showing; (c) shrunken glomerulus (g) with congested capillaries (Gc), few dense shrunken nuclei of mesangial cells (red arrowheads), surrounded by unpreserved Bowman's capsule (black arrow), and wide renal space (*). (d) Proximal (p) and distal (d) tubules lined with shrunken cells with some condensed (n) and exfoliated nuclei (red arrow). The diabetic group treated with insulin (e & f) and REO (g & h) showing (e & g) apparently normal glomerulus (g) with congested capillaries (Gc) and normal nuclei (n) of mesangial cells, surrounded by Bowman's capsule (black arrow) with normal Bowman's space (*). REO-treated rats show some enlarged darkly stained nuclei (red arrowhead) of mesangial cells. (f & h) Distal tubule (d) shows some nuclear derangement with desquamated nuclei inside the tubular lumen (red arrows); some show exfoliated, darkly stained, and small nuclei (n). Some proximal tubules (p) show exfoliated cells (Δ) and no brush border. (i-j) Diabetic rat treated with insulin & REO showing (i): normal glomerulus (g) enclosing glomerular capillaries (Gc) and normal nuclei (red arrowheads) of mesangial cells surrounded by preserved Bowman's capsule (black arrow). (j): Distal tubule (d) showing areas of destructed cells (Δ), proximal tubules (p) appear normal, congested blood capillaries (Bc) are detected between tubules (Toluidine blue, x1000).

Toluidine blue stained sections from control rats showed normal renal corpuscles, including glomerular capillaries and mesangial nuclei, and surrounded by a Bowman capsule lined with a single layer of flattened cells on its parietal layer (Figure 4a). Proximal tubules showed small lumens bordered with pyramidal cells with dark cytoplasm, basal vesicular nuclei, and a well-developed brush border. The distal tubules have cubical cells with rounded vesicular nuclei, lighter cytoplasm, and less prominent brush borders (Figure 4b). Sections of the diabetic rat's kidney showed shrunken and distorted glomeruli containing congested glomerular capillaries with few dense shrunken mesangial cell nuclei. Unpreserved Bowman's capsule surrounds glomeruli with tiny, flattened nuclei and wide urinary space (Figure 4c). Both proximal and distal tubules displayed degeneration with ill-defined lumens. Lack of linear nuclear organization with shrunken, condensed, and exfoliated nuclei and loss of brush border were seen in the cells lining the renal tubules (Figure 4d). 

When insulin or REO was administered to diabetic rats, a greater proportion of renal glomeruli with normal architecture was detected. These glomeruli contained normal mesangial cell nuclei and normal Bowman's capsules lined with a single layer of flattened cells (Figure 4e, 4g). In both groups, the distal tubules' linear nuclear organization was disrupted, with some nuclei sloughed into the lumen. In contrast to the REO-treated group, which exhibited normal proximal tubules, the insulin-treated rats displayed pale, exfoliated columnar cells with disrupted brush borders (Figure 4f, 4h). Sections from diabetic rats treated with REO and insulin demonstrated kidney architecture similar to the control groups, where most glomeruli and tubules seemed normal (Figure 4i, 4j).

PAS Staining 

**Figure 5:**
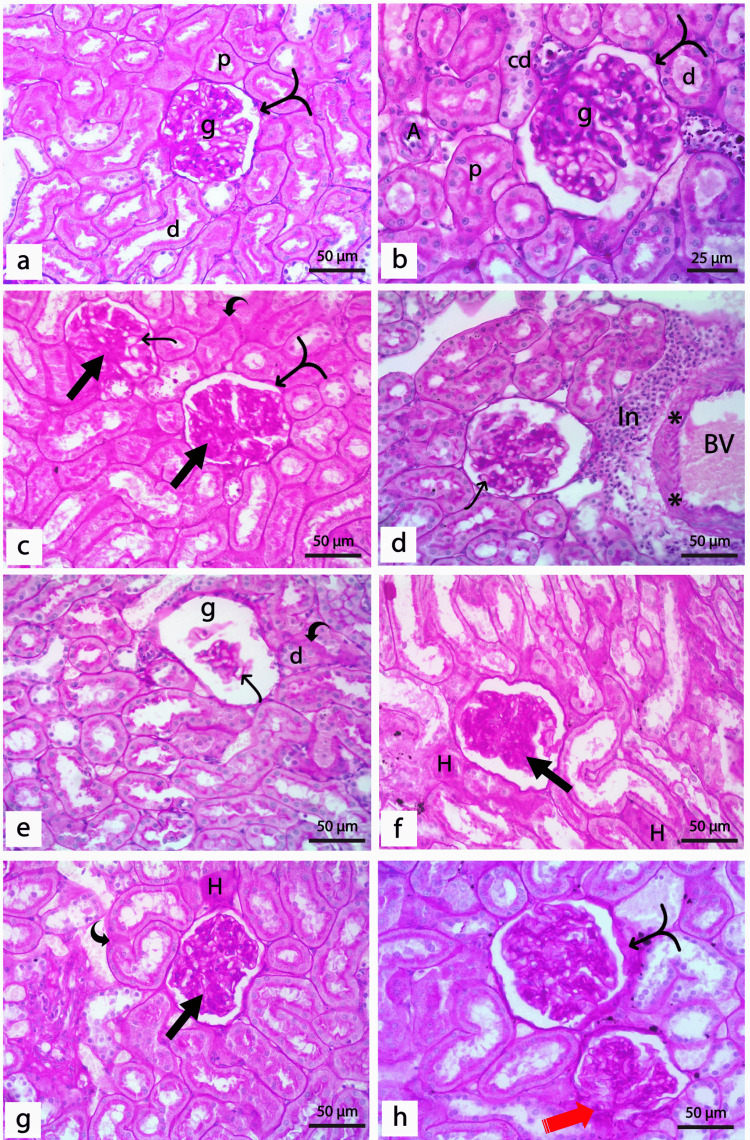
Sections of PAS-stained renal cortex of the control and diabetic groups (a & b) The control group showed a positive reaction for PAS in the glomerular capillaries basement membrane and adjacent mesangial cells (g) with thin basement membranes of the Bowman's capsule (split arrow). Proximal (p) and distal (d) tubules demonstrate a normal thin basement membrane and positive PAS reaction in the brush border. Moreover, a normal PAS staining of the collecting ducts (cd) and normal intimal thickening of blood arteriole (A) are detected. (c-h) diabetic group showing; (c&d): Glomeruli show diffuse mesangial sclerosis (black arrow), thickened glomerular capillary basement membranes (thin curved arrow), thickened parietal layer of Bowman's capsule (split arrow), thickened tubular basement membrane (thick curved arrow), the blood vessels (BV) show intimal fibrosis (*) and surrounded by excess inflammatory infiltrates (In). (e) The glomerulus (g) shows degenerated tuft of capillaries with thickened basement membrane (thin curved arrow) and thickened tubular basement membranes (thick curved arrow) of proximal (p) and distal (d) tubules. (f) The glomerulus shows a Kimmelstiel-Wilson nodule (black arrow) and tubular hyalinosis (H). (g) Glomerulus showed diffuse sclerosis (black arrow), tubular hyalinosis (H), and very thickened walls of both venules (V) and arterioles (A). (h) The glomerulus shows hyalinosis at the vascular pole of the renal corpuscle (red arrow) and thickened parietal layer of Bowman's capsule (split arrow). (a, c-h: PAS x400, bar 50μm; b PAS × 600, bar 25μm).

Cortical parenchyma of the rat kidney of the control group demonstrated a normal positive PAS reaction in the mesangium and basement membranes of glomerular capillaries, Bowman's capsules, proximal tubules, distal tubules, and collecting ducts. The brush border of proximal and distal tubules was also positive for PAS, while blood arterioles showed normal intimal thickening (Figure 5a, 5b). Sections from diabetic rats demonstrated a strong positive PAS reaction in the glomerulus, which shows diffuse mesangial sclerosis, thickened glomerular capillary, and Bowman's capsules basement membranes. Some glomeruli show they degenerated capillary tufts with the thickened basement membrane, while others show Kimmelstiel-Wilson nodules. Thickened basement membranes of renal tubules, tubular hyalinosis, and thickened walls of blood vessels surrounded by excess inflammatory infiltrate were also detected in the diabetic group (Figures 5c-5h). 

**Figure 6:**
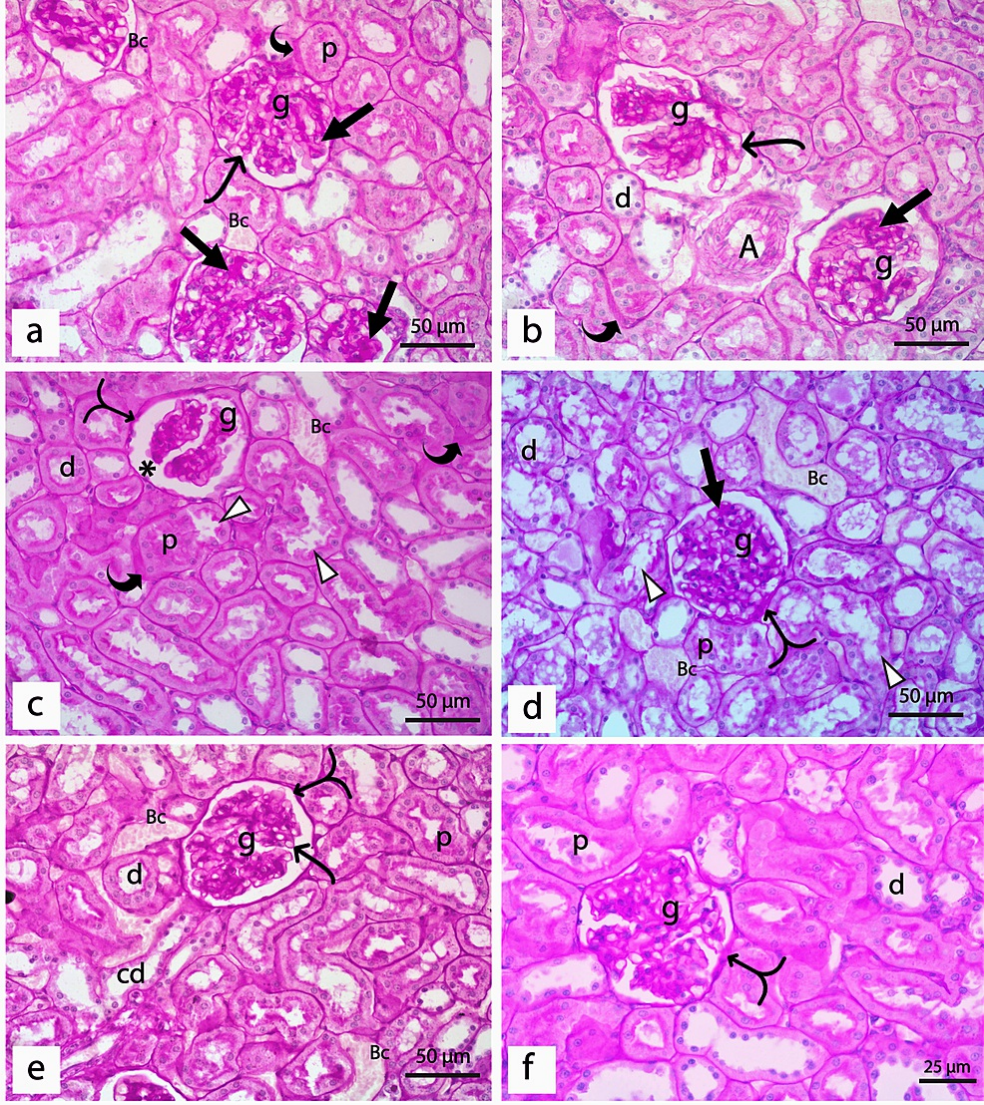
Sections of PAS-stained renal cortex of treated groups. (a&b) Diabetic rats treated with insulin showing the glomeruli (g) with thickened glomerular capillary basement membrane (thin curved arrow), mesangial sclerosis (black arrow), and thickened wall of proximal (p) and distal tubules (d) (thick curved arrow). Notice the increased intimal thickness of the blood arteriole (A). (c&d)Diabetic rats treated with REO showing (c) bifid glomerulus (g) with wide Bowman's space (*) and areas of thickened Bowman's capsule (split arrow). The distal tubules (d) show thin and intact walls, but the proximal tubule (p) shows areas with thickened basement membrane (thick curved arrow) and disrupted brush border (Δ). (d): Normal glomerulus (g) with slight mesangial expansion (black arrow) and areas of thickened Bowman's capsule (split arrow), as well as some proximal (p) and distal (d) tubules, show disrupted brush border (Δ). Notice the congested blood capillaries (Bc) between the tubules. (e&f) Diabetic rats treated with insulin and REO showing (e) Glomerulus (g) show normal positive thin glomerular capillary walls (thin curved arrow) and thin basement membrane of the Bowman's capsule (split arrow), a thin wall of proximal (p) and distal tubules (d), thickening of the basement membrane of the collecting duct (cd), and congested blood capillaries (Bc). (f) The glomerulus shows a little thickened Bowman's capsule (split arrow) and a normal thin basement membrane of the proximal (p) and distal (d) renal tubules. (a-e: PAS × 400, bar 50μm; f: PAS × 600, bar 25μm).

The diabetic group treated with insulin displayed a moderate to strong positive PAS reaction where the glomeruli with thickened capillaries walls, mesangial sclerosis, and increased intimal thickness of blood arterioles were detected. Proximal and distal tubules showed thickened basement membranes (Figures 6a, 6b). For diabetic rats treated with REO, histological examination of renal cortex stained with PAS revealed normal glomeruli with slight mesangial expansion and thickened Bowman's capsule patches. Some bifid glomeruli with wide Bowman's space were also detected. Furthermore, the distal tubules displayed a thin intact basement membrane. They disturbed the brush border, whereas the proximal tubules displayed thickened areas of the basement membrane with a damaged brush border (Figures 6c, 6d). The combined treated group with both insulin and REO demonstrated marked improvement in the structure of the glomeruli and renal tubules, which demonstrated normal positive PAS reaction. Notably, a few glomeruli still showed little patches of thickened Bowman's capsule (Figures 6e, 6f). 

We further assessed the degree of sclerosis by measuring the percentage of PAS-positive staining in the glomeruli of different experimental groups, as shown in Table 3. Our results demonstrated a statistically significant increase (p < 0.001) in the glomerulosclerosis index in the diabetic group when compared with the control. Specifically, treatment with insulin, REO, or both combined markedly reduced the sclerotic area in the glomeruli as a highly significant difference (p < 0.001) was detected compared to the diabetic rats. It is worth mentioning that the combined administration of insulin and REO resorted to the normal glomerulosclerosis index of the kidney.

Immunohistochemical staining

BAX Immunostaining

**Figure 7:**
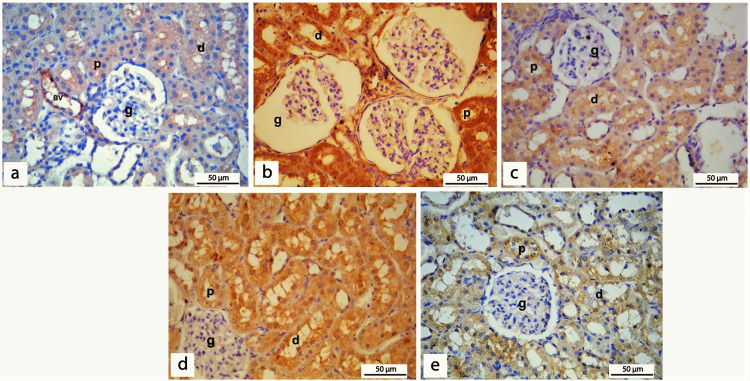
BAX immunohistochemical staining of kidney sections of different experimental groups. (a) Renal cortical tissue of the control group shows very weak BAX immunostaining in the cytoplasm of the cells lining the proximal (p), distal tubules (d), and in the endothelium of blood vessels (BV); however, no immune-reactive cells are detected in the glomeruli (g). (b) Sections of the diabetic group show strong diffuse BAX immunostaining in the cytoplasm of the cells lining the proximal (p) and distal (d) tubules. At the same time, weak staining is detected in the cytoplasm of a subset of cells forming the glomeruli (g). (c) Section of diabetic rats treated with insulin showing moderately stained immune-reactive cells of proximal (p) and distal (d) tubules. (d) Section of diabetic rats treated with REO shows moderately stained immune-reactive cells of proximal (p). However, some distal (d) tubule cells detect stronger staining than insulin. (e) Section of diabetic rats treated with insulin and REO shows mild BAX immunostaining of the cytoplasm of cells lining the proximal (p) and distal (d) tubules with no staining in the cells forming the glomeruli. (Immunohistochemical staining of BAX, x400).

BAX, a well-known marker of apoptosis, was evaluated by immunohistochemistry to assess the impact of REO on apoptosis in the diabetic nephropathy rat model. In the present study, IHC staining of sections from rats of the control group showed that BAX immunostaining of renal cortical tissue was mainly observed as very weak staining in the cytoplasm of the cells lining the renal tubules and in the endothelial lining of blood vessels (Figure 7a). Sections of diabetic rats showed strong diffuse BAX immunostaining in the cytoplasm of cells lining the proximal and distal tubules. At the same time, weak staining is detected in the cytoplasm of a few cells of the glomeruli. In the diabetic group, BAX expression was statistically higher (p < 0.001) than in the control group (Figure 7b). Insulin or REO administration in diabetic rats was able to induce a highly significant reduction (p < 0.001) of BAX expression compared to diabetic groups (Figures 7c, 7d). The concomitant administration of insulin and REO induced a highly significant reduction (p < 0.001) of BAXexpression, as evidenced by diminished immunostaining of BAX in the cytoplasm of cells lining the renal tubules (Figure 7e) (Table 4). These findings indicated a potential anti-apoptotic effect of insulin and REO in STZ-induced diabetic nephropathy, which may be attributed to targeting BAX expression. Of note, combining insulin with REO has a more significant impact on reducing BAX expression than either treatment alone.

Proliferating cell nuclear antigen (PCNA) immunostaining

**Figure 8:**
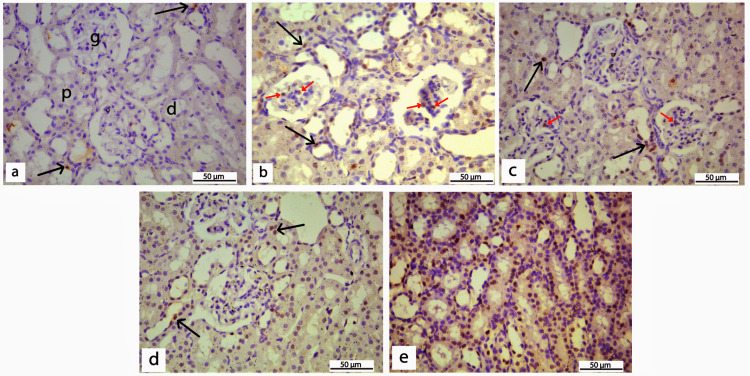
PCNA immunohistochemical staining of kidney sections of different experimental groups. (a) Renal cortical tissue of the control group showing no PCNA expression in the glomeruli (g) with only a few cells lining the tubules (black arrow) showing weak positive expression. (b) Section of the diabetic group showing an increase in the number of PCNA immunoreactive cells in both glomeruli (red arrows), proximal (p), and distal (d) tubules (black arrows). (c) Section of diabetic rats treated with insulin shows a moderate number of cells with strong PCNA immunostaining in proximal (p), distal (d) tubules, and interstitial cells (arrowheads) with very few positive cells inside the glomeruli (red arrow). (d) Section of diabetic rats treated with REO shows strong PCNA immunostaining (black arrow) in cells lining both proximal (p) and distal (d) tubules and interstitial cells (arrowheads). (e) Section of diabetic rats treated with insulin and REO showing strong positive expression of PCNA in the majority of cells lining the proximal (p), distal (d) tubules, and collecting duct (cd). (Immunohistochemical staining of PCNA, x400).

In the control group, PCNA immunohistochemical staining of renal cortical tissue was limited to the nuclei of a small number of cells lining both proximal and distal tubules (Figure 8a). When compared to the control, a section of diabetic rats revealed a substantial increase (p < 0.001) in the number of PCNA immunoreactive cells in the glomeruli and tubules with positive but weak to moderate PCNA expression (Figure 8b). When insulin or REO was administered to diabetic rats, the number of cells with strong PCNA immunostaining in proximal and distal tubules, interstitial cells, and peri-glomerulus increased significantly compared to the control (Figures 8c, 8d). Notably, there were very few or no PCNA-positive cells in the glomeruli of these two groups. Importantly, diabetic rats treated with insulin significantly increased PCNA expression (p < 0.05) compared to diabetic untreated rats. However, diabetic rats treated with REO did not show any significant difference when compared with diabetic rats. The diabetic group treated with both insulin and REO revealed that PCNA is strongly expressed in a large number of cells lining the proximal and distal tubules and collecting ducts, which was significantly different from both the control and untreated diabetic group (p < 0.001) (Table 4). Of note, no PCNA expression was detected in the glomeruli of the combined treated group (Figure 8e).

**Table 4:** Semiquantitative scoring of immunohistochemically stained sections of different experimental groups REO: Rosemary Essential Oil, BAX: Bcl-2 Associated X-protein, PCNA: Proliferating cell nuclear antigen. Compared to ^a^control, ^b^REO control, ^c^diabetic, ^d^diabetic insulin-treated,^ e^diabetic REO-treated, ^f^diabetic insulin & REO-treated, *p < 0.05, **p < 0.01

Animal groups	Average score of BAX Mean ± SD	Average score of PCNA Mean ± SD
Negative control	3.1 ± 0.74	1.9 ± 0.32
REO positive control	3.5 ± 1.71	1.8 ± 0.63
Diabetic	7.5 ± .072^ **(a,b,d,f) *(e)^	3.9 ± .074^ **(a,b, f) ,*(d)^
Diabetic insulin-treated	5.2 ± 0.63 ^**(a,b,c^^( ^^, *(f)^	4.8 ± 0.63 ^**(a,b,f^^(^^, *(c)^
Diabetic REO-treated	6.1 ± 0.99^ **(a,b, f), *(c)^	4.6 ± 0.84^ **(a,b, f) ^
Diabetic insulin & REO treated	3.67 ± 0.71 ^**(c, f), *(d)^	6.8 ± 0.63 ^**(a,b,c, d,f)^

## Discussion

There is emerging evidence that REO can help ameliorate certain illnesses, but little research has focused on its nephroprotective properties [10-12]. So far, the possible protective role of REO in diabetic nephropathy is not yet well understood. Therefore, the current research assessed REO's potential benefits on the biochemical, histological, and immunohistochemical alterations of diabetic nephropathy and compared its effects to insulin or their combination in male albino rats. 

Hyperglycemia, associated with diabetes, is a crucial risk factor for diabetic nephropathy. So, we decided to assess the effect of REO alone or combined with insulin in controlling diabetic rats' blood glucose levels. Our results demonstrated that REO significantly reversed the elevated levels of blood glucose detected in the diabetic group; however, a significantly high level was still detected when compared to the control group. These findings align with previous studies that demonstrated the anti-hyperglycemic effects of rosemary extracts and many of their constituents [27-30]. REO's anti-hyperglycemic and insulin-like effects can be attributed to several mechanisms, including its action to enhance glucose uptake and consumption, decrease glycogen content and increase glycolytic rate in hepatocytes and muscle cells [28,29], as well as its ability to inhibit glucose production and gluconeogenesis in liver cells [31]. In addition, based on several in vitro studies, the whole rosemary extract and many of its ingredients displayed dose-dependent inhibitory actions against essential enzymes controlling blood glucose levels, including pancreatic amylase [32], pancreatic lipase, hormone-sensitive lipase [33], and α-glucosidase [34]. Of note, our results revealed that the effect of insulin administration outperforms REO alone in controlling diabetic rats' blood glucose levels.

Moreover, combined insulin and REO were more effective than administering either one alone in lowering the blood glucose level of diabetic rats. This can be attributed to combining distinct mechanisms of actions of REO and insulin in regulating blood glucose levels. Further investigations are needed to assess the potential benefits of REO if used as supplementary to insulin in diabetic patients. Our results demonstrated that STZ administration significantly increased the levels of assessed renal excretory function tests compared with the control group, supporting earlier research [35-37]. The high serum urea, creatinine, and uric acid levels and detected hyperglycemia in our experiment indicate renal tissue injury. Diabetic rats treated with REO or insulin had lower serum urea and uric acid levels than the diabetic rats; however, the levels were significantly higher than those of the control group. In line with our results, Najla (2012) determined that therapy with rosemary extract significantly reduced the rise in blood urea nitrogen, serum uric acid, and creatinine in diabetic rats [38]. Our results imply that although REO, insulin, or combined with insulin could ameliorate the effect of STZ on kidney function, none of them could restore any of the serum urea, creatinine, and uric acid to their normal levels.

It is well-established that oxidative stress, regulated by enzymatic and non-enzymatic mechanisms, plays a pivotal role in diabetes-related complications. It has been previously reported that the STZ-induced diabetic rat model exhibits aberrant levels and activities of oxidative stress markers such as elevated MDA, reduced GSH levels, and decreased SOD and CAT activity [39,40], which is comparable with the current study findings. However, one contradicting point detected in our data was the increased activity of CAT in the kidney of rats after STZ administration. On the other hand, two previous studies supported our findings by reporting an increase in CAT antioxidant enzymatic activity in hepatocytes and erythrocytes of STZ-induced diabetic rats [41,42]. These contradicting findings might be attributed to variations in doses and durations of STZ administration, tissue specificity, and other experimental conditions. One might assume that the increased catalase activity in the kidney of diabetic rats may represent a compensatory or adaptive response to increased oxidative damage in diabetic nephropathy. 

The present work revealed that REO demonstrated high antioxidant activity in vitro, as determined by the DPPH scavenging assay, with an IC50 value of 78.4 μl/ml. Consistent with our data, previous studies using the same or different in vitro antioxidant assays reported the high antioxidant activity of REO [14,43]. However, variation of IC50 values between different studies can be explained by the lack of standardized experimental conditions, such as using different doses or formulations of REO, which may have different chemical compositions. Our results consistently demonstrated that the administration of REO significantly reversed all STZ-induced perturbation of the antioxidant markers in the kidney tissue. For diabetic rats treated either with REO alone or combined with insulin, our findings demonstrated a substantial reduction in the level of MDA in comparison to the diabetic group. This data suggests that REO can reduce lipid peroxidation and preserve cellular integrity, which might be owing to its capacity to eliminate free radicals, as detected by the DPPH test.

Furthermore, separate and simultaneous administration of REO and insulin-induced a significant increase in the level of GSH (non-enzymatic antioxidant), increased the activity of SOD and decreased the activity of CAT antioxidant enzymes compared with the diabetic rats. Our findings are supported by earlier investigations showing REO's antioxidant properties [44,45]. The antioxidant activity of REO can be elucidated by Nieto et al. findings, who reported that rosemary's antioxidant activities are attributable to its high amount of isoprenoid quinones, which are powerful ROS chelators and peroxyl radical scavengers [46]. Another possible mechanism that might explain the antioxidant activity of REO is its previously reported ability to reduce the oxidation of DNA bases and single-strand DNA breaks [47]. It is worth noting that combining REO with insulin had a greater antioxidant impact on kidney tissue than using REO or insulin alone.

Results of H&E and toluidine blue-stained sections of diabetic rats demonstrated shrunken and disrupted glomeruli enclosing wide and congested glomerular capillaries with few dense shrunken nuclei of mesangial cells. Varying degrees of tubular degeneration and necrosis were detected in the renal cortex, where both proximal and distal tubules showed ill-defined lumens, shrunken, condensed, and exfoliated nuclei, as well as loss of brush borders. These findings are in agreement with that of previous studies [48,49]. The detected structural atrophic changes in the glomeruli and congested glomerular capillaries of diabetic rats can be attributed to sustained hyperglycemia which causes hyperfiltration and vasodilatation [50,51]. Moreover, hyperglycemia induces protein kinase C (PKC), which boosts PGE1 synthesis and activates the vascular endothelial growth factor (VEGF), all of which result in albuminuria, endothelial dysfunction with subsequently increased permeability and glomerular hyperfiltration [52,53].

On the other hand, the detected tubular damage in the present study can be justified by the fact that the persistent hyperglycemia in diabetic rats caused an increase in activin A expression, a member of the transforming growth factor-β (TGF-β) protein family in the renal tubules lining epithelium [54]. A high level of activin A induces fibronectin production through the Smad signaling pathway with subsequent stimulation of tubulointerstitial fibrosis [55,56]. Furthermore, intracytoplasmic hyaline droplet deposition in the cells lining the renal tubules causes cellular degeneration with subsequent renal tubular degeneration and regeneration [57].

Examination of PAS-stained sections of the renal cortex of diabetic rats showed diffuse mesangial sclerosis, thickened glomerular capillary and Bowman's capsules basement membranes, proximal and distal tubules basement membranes thickening, tubular hyalinosis, and thickened walls of blood vessels together with a large number of inflammatory cells between the renal tubules. These findings agree with Soetikno et al. and Mestry et al. [48,58]. Mesangial sclerosis and thickening of basement membranes of various renal components can be attributed to reduced activities of matrix metalloproteinases (e.g., MMP2 and MMP9) and glomerular proteinases (e.g., cathepsin B and collagenase), as well as overproduction and deposition of glycogen in the renal tubules and Bowman's capsules of diabetic rats [59,60]. In addition, elevated Megsin, a member of the protease inhibitors family, inhibits the activities of plasmin and MMP and promotes progressive mesangial growth [60]. It is worth mentioning that structural alterations in the renal glomeruli and tubules might explain the elevated levels of serum urea, creatinine, and uric acid detected in our study on diabetic rats [61].

Although a growing body of evidence using different experimental models emphasized the anti-hyperglycemic effects of REO, it is yet unknown if REO improves the histological alterations of diabetic nephropathy. To our knowledge, the current work is the first to present the nephroprotective effects of REO in STZ-induced diabetic nephropathy based on the histological analysis of renal tissues. Our findings from the histological assessment of diabetic rats treated with insulin or REO showed that STZ-induced impact on the kidney tissue was partially corrected, with a greater proportion of glomeruli displaying normal architecture; nevertheless, congested glomerular capillaries were still detected. Furthermore, both groups improved the proximal and distal tubular degeneration caused by STZ. Interestingly, examination of kidney sections from diabetic rats treated with REO and insulin revealed that the renal architecture was nearly identical to that of controls, with only some cells in a few distal tubules being disrupted. Our findings from the histopathological examination were in line with that of Hassanen and his coworkers, who reported the protective effects of rosemary against diethylnitrosamine-induced renal injury in rats [62]. The structural changes detected after the administration of REO can be attributed to its antioxidant and anti-hyperglycemic effects previously discussed. In addition, it has been reported in many previous studies that the inhibitory action of REO on many pro-inflammatory mediators, such as decreased PGE2, MMP2, NO, and tumor necrosis factor-alpha (TNF-alpha) [63,64]. Carnosol, one of the main components of the rosemary, acts by inhibiting proliferation and modulation of epithelia-mesenchymal transition, which can attribute to the nephroprotective effects of REO [65]. 

BAX, a well-recognized marker of apoptosis, is a pro-apoptotic protein that belongs to the Bcl-2 gene family. Similar to our findings, previous studies demonstrated a marked increase in BAX expression in cells lining glomeruli and renal tubules of diabetic rats using immunohistochemical analysis [66,67]. Previous research has shown that hyperglycemia causes mesangial cell death via oxidative stress and inflammation, marked by an increase in the BAX/Bcl-2 ratio [68,69]. Diabetes-induced oxidative stress causes mitochondrial DNA mutations, leading to mitochondrial dysfunction and an increase in the formation of ROS with subsequent cell death [4]. On the other hand, our results revealed that BAX expression decreased after separate and concomitant administration of REO and insulin; however, combined administration showed lower expression of BAX. These findings indicate the anti-apoptotic activity of REO in renal tissue, which opposes that of Dorrie et al. 2001 who reported the pro-apoptotic effects of carnosol, a component of rosemary in B-lineage leukemia cells [70]. This contradiction can be explained by using different experimental models, different formulations, doses of rosemary, and different assessment tools. Further analysis will be crucial to solve this contradiction and assess REO's role in regulating apoptosis in diabetic nephropathy.

PCNA is a nuclear protein essential for DNA replication and is considered one of the critical markers of cell proliferation. In the current study, the examination of PCNA-stained sections showed more PCNA-positive cells in diabetic rats' renal cortex compared to the control, fully in line with the results of different previous studies, which demonstrated increased PCNA expression in STZ-induced diabetic rats when compared to negative control [71,72]. Moreover, the PCNA positive cell count was significantly higher in the insulin-, REO-, or combination-treated groups than in the diabetes group. Notably, most PCNA immunopositive cells were detected in the tubules and the interstitium, with very few or no PCNA-positive cells in the glomeruli, which agrees with the findings of Young et al. [72]. The current study's findings highlighted the potential anti-proliferative activity of REO and insulin on the glomerular cells and the proliferation-stimulating activity on the tubular and interstitial cells. Earlier in vitro studies on different types of malignant cell lines demonstrated that the anti-proliferative activity of REO was attributed to its modulation of the survival pathways of protein kinase B (Akt) and mammalian target of rapamycin (mTOR) [73], as well as through induction of growth arrest and cell cycle block [74]. On the other hand, the ability of rosemary extract to induce proliferation has been reported in two different in vitro studies using human adipose stem cells and splenic mononuclear cells [75,76]. More investigations are needed to understand how REO affects the proliferation of various kidney tissue components and to learn more about the underlying molecular mechanisms and related pathways.

The present study's primary limitation was the lack of a multi-dose design (low, medium, and high dosage) of REO to examine the possible dose-effect relationships, which might be addressed in future studies. Moreover, a study of longer duration is needed to assess the effects of prolonged REO therapy on kidney function and possible side effects or toxicities. There is also a need for further research to validate our findings and explore how REO can affect various mechanisms implicated by diabetes in the kidneys. Moreover, it will be interesting to assess the effect of REO administration on different organs.

## Conclusions

This study revealed that although REO demonstrated potential protective effects on kidney structure, it could not restore normal renal function. Our results confirmed several positive actions of REO, which were demonstrated by its antioxidant, anti-inflammatory, anti-apoptotic, pro-proliferative, and minimal anti-hyperglycemic actions, all of which help reduce the harmful actions of diabetes in the kidney. We also detected the protective effect of insulin on renal tissues, which are already used for the clinical treatment of diabetic patients. Although the promising nephroprotective roles of REO were detected in the current experiment, we showed that the combined administration of insulin and REO has a more potent impact in ameliorating the diabetic effects on the kidney. Combined insulin and REO administration can restore the normal range of blood glucose, renal excretory function tests, the enzymatic and non-enzymatic antioxidant markers, and the histological structure of the renal cortex. Hence, data presented in the current study indicated that combined administration of insulin and REO has more nephroprotective effects than either administered individually. More studies are warranted further to delineate the mechanisms of action of REO in diabetes and to validate these findings in humans.

## Figures and Tables

**Figure 9:**
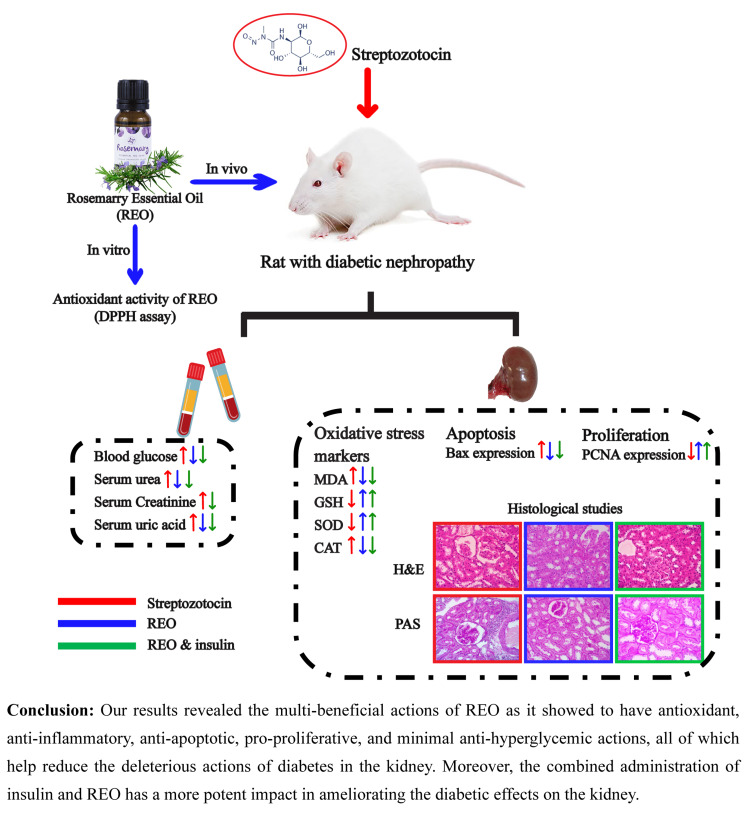
Graphical abstract
